# ‘To know before hand is to freeze and kill’ Commentary on… Should psychiatrists write fiction?

**DOI:** 10.1192/bjb.2017.22

**Published:** 2018-04

**Authors:** Daniel Racey

**Affiliations:** 1 ST4 Child and Adolescent Psychiatry, The Terraces, Mount Gould Hospital, Livewell Southwest, Plymouth PL4 7QD, UK

## Abstract

In this article I argue that fictional accounts of mental illness should be unethically unobliged. I suggest that art is not generated with conscious ethical intent and provide evidence that art proceeding from an ethical agenda is more likely to be poor art. I also consider ways in which a writer-doctor might need to compromise what they articulate to maintain a professional ethical integrity.

**Declaration of interest:**

None.

For only after, can one nail down, examine, explain. To try to know beforehand is to freeze and kill. Self-consciousness is the enemy of all art, be it acting, writing, painting, or living itself, which is the greatest art of all.

Ray Bradbury^1^

Beveridge^2^ argued that fiction supports psychiatrists to imaginatively enter other lives to become more ethical and empathetic. The previous article^3^ changes the slant: psychiatrist/writers should enter the life of readers to encourage them to become more ethical and empathetic regarding mental health. Oyebode, a poet/psychiatrist, sees literature as lacking conscious ethical intent, but argues that a secondary outcome of fictional investigations of psychiatry is to influence how society sees mental disorder and how politicians will tackle this area.^4^

Will Self has written many pieces of fiction with a recurring psychiatrist character, Zack Busner, who sometimes resembles Ronnie Laing, sometimes Oliver Sachs. For Self, psychiatry has become central to his writing, because:

*… psychiatrists stand – whether they acknowledge it themselves […] at the threshold between happiness and sadness and between sanity and madness […] I'm thinking […] in terms of priests who manage the transition from the phenomenal to the numinal.*^5^

In a secular age dominated by scientism, psychiatrists function as meaning-makers. Given this status, shouldn't psychiatrists who write show an ethical loyalty to the project of psychiatry?

The way in which creativity proceeds and how art and psychiatry interact with each other can be approached using the framework of one of psychiatry's foundational figures – Karl Jaspers. Jaspers identified two modes of knowing, which should weave together in the work of a psychiatrist: Verstehen and Erklären.^6^ Erklären tries to make explanatory sense of phenomena by finding the laws that govern them. The psychiatrist who engages in Verstehen tries to make empathetic sense of phenomena by looking for the perspective from which the phenomenon appears to be meaningful. Jaspers is describing the art and science of medicine, insisting that scientific explanations are necessary but not sufficient accounts of our patients.

Fiction attempts to subjectively understand the human condition – it is Verstehen. At the same time, fiction is not just a right-brained tapping of the unconscious. A writer gains by having authority over the subject matter: to be coherent, to be up-to-date, to teach us, to please us with erudition. We know the difference between well-researched and poorly researched novels. However, writing that is overly occupied by Erklären will fall dead from the womb.^7^ A Pulitzer Prize winner writes:

*‘What's erroneous is the assumption that the thoughtful analysis and willful insertion of that in the work is the creative process […] it's the antithesis of the process […] If you start perverting that with other motives to write, your ability to become an artist is severely hampered, if not destroyed.’* ^8^

In the accompanying piece, the authors simplify and rationalise the writing process, which they depict as ‘*Firstly, the writer gathers information through research. Secondly, a story framework, however loose or rigid that may be, is devised. Thirdly, a coherent narrative is constructed through putting the words onto paper.*’^9^ This strikes me as true when I write professionally or academically. I would suggest that the process of writing fiction is fundamentally different, that the authors misunderstand the writing process by assuming the ego's fingers have a firm grip of the pen. Numerous writers have described the creative process as passive,^8^^,^^9^ for example, Paul Bowles states:

If I am writing fiction, I am being invented. I cannot retain any awareness of identity. The two states of being are antithetical. The author is not at a steering wheel […]^9^

The creating artist may be caught in an unconscious dream, but Margaret Atwood does not allow so easy an abdication of moral responsibility:

Why do authors wish to pretend they don't exist? It's a way of skinning out, of avoiding truth and consequences.^10^

Ray Bradbury states: ‘*For only after, can one nail down, examine, explain*.’^1^ This feels true to the writing experience: that creation is often unconscious, but the subsequent shaping of the material is conscious and where ethics become relevant. The poet Selima Hill describes the moral editorial step which occurs late in her creative process. She uses the following rubric to guide her:

*[…] the morals of the thing. Is it libellous? Is anyone's reputation going to suffer? […] Am I exploiting someone else's work, or life? Or might it, on the other hand, have a positive (morally ‘good’) effect?*^11^

The power dynamic in psychiatry is so asymmetric and the potential consequences of breaching confidentiality are so severe that when psychiatrists write about psychiatry, our policing needs to be rigorous. How we achieve this is another topic in itself.

Selima Hill concludes that the function of art is not that of Erklären but the subversion of Erklären.

*It is not the place of art to draw conclusions or even to understand. On the contrary, isn't the whole point that it is non-judgemental? That it undermines the making of value judgements? It is modest, helpless, useless, but at the same time determined, and just. I think of Heaney's phrase ‘to set the darkness echoing’ – where darkness is what it is, and we can neither flinch nor sink.*^11^

Hill is arguing that art should be ambiguous, should avoid conclusions. Psychiatry is used to shades of grey in its epistemology, nosology and ethics. More than other branches of medicine, we require negative capability – the skill of sitting with uncertainty without irritably reaching after a simplistic explanation.^12^ This is Verstehen – a different kind of understanding, one that does not make value judgements. To achieve such an end, the artist/psychiatrist will put aside Erklären and ethics. An activity that is ‘*modest, helpless, useless but just and determined […] where […] we […] neither flinch nor sink*’ sounds like what most psychiatrists do, the kind of modest psychiatry we can believe in, the psychiatry that we actually practise in shabby clinics and battered wards ‘*where we set the darkness echoing’*.

## About the author

**Dr Daniel Racey**, ST4 Child and Adolescent Psychiatry, The Terraces, Mount Gould Hospital, Livewell Southwest, Plymouth, PL4 7QD, UK.
